# The value of joint ultrasonography in predicting arthritis in seropositive patients with arthralgia: a prospective cohort study

**DOI:** 10.1186/s13075-018-1767-9

**Published:** 2018-12-19

**Authors:** Marian H. van Beers-Tas, Annelies B. Blanken, Mark M. J. Nielen, Franktien Turkstra, Conny J. van der Laken, Marlies Meursinge Reynders, Dirkjan van Schaardenburg

**Affiliations:** 1 Amsterdam Rheumatology and immunology Center | Reade, Dr. Jan van Breemenstraat 2, 1056 AB Amsterdam, The Netherlands; 2 Netherlands Institute for Health Services Research (NIVEL), Otterstraat 118-124, 3513 CR Utrecht, The Netherlands; 3 Amsterdam Rheumatology and immunology Center, VU University Medical Center, De Boelelaan 1118, 1081 HZ Amsterdam, The Netherlands; 4 Amsterdam Rheumatology and immunology Center | Academic Medical Center, Meibergdreef 9, 1105 AZ Amsterdam, The Netherlands

**Keywords:** Ultrasonography, Synovial thickness, Power Doppler, Arthralgia patients, Arthritis, Seropositive, Autoantibodies

## Abstract

**Background:**

The value of joint ultrasonography (US) in the prediction of clinical arthritis in individuals at risk of developing rheumatoid arthritis (RA) is still a point of debate, due to varying scanning protocols and different populations. We investigated whether US abnormalities assessed with a standard joint protocol can predict development of arthritis in seropositive patients with arthralgia.

**Methods:**

Anti-citrullinated protein antibodies and/or rheumatoid factor positive patients with arthralgia, but without clinical arthritis were included. US was performed at baseline in 16 joints: bilateral metacarpophalangeal 2–3, proximal interphalangeal 2–3, wrist and metatarsophalangeal (MTP) joints 2–3 and 5. Images were scored semi-quantitatively for synovial thickening and for positive signs on power Doppler (PD). Association between US abnormalities and arthritis development at the joint and at the patient level was evaluated. Also, we investigated the added value of US over clinical parameters.

**Results:**

Out of 163 patients who underwent US examination, 51 (31%) developed clinical arthritis after a median follow-up time of 12 (interquartile range 5–24) months, of which 44 (86%) satisfied the 2010 American College of Rheumatology/European League Against Rheumatism classification criteria for RA. US revealed synovial thickening and PD in at least one joint in 49 patients (30%) and 7 patients (4%), respectively. Synovial thickening was associated with both development and timing of clinical arthritis in any joint (patient level) when MTP joints were excluded from the US assessment (odds ratio 6.6, confidence interval (CI) 1.9–22), and hazard ratio 3.4, CI 1.6–6.8, respectively, with a mean time to arthritis of 23 versus 45 months when synovial thickening was present versus not present). There was no association between US and arthritis development at the joint level. Predictive capacity was highest in the groups with an intermediate and high risk of developing arthritis based on a prediction rule with clinical parameters.

**Conclusions:**

Synovial thickening on US predicted clinical arthritis development at the patient level in seropositive patients with arthralgia when MTPs were excluded from the US assessment. Positive PD signs were infrequently seen in these at-risk individuals and was not predictive. In patients at intermediate risk of RA, US may help to identify those at higher risk of developing arthritis.

**Electronic supplementary material:**

The online version of this article (10.1186/s13075-018-1767-9) contains supplementary material, which is available to authorized users.

## Background

Rheumatoid arthritis (RA) is a chronic autoimmune disease that is characterized by synovial inflammation and swelling. In the at-risk phase before clinical RA development, the presence of autoantibodies such as anti-citrullinated protein antibodies (ACPA) and/or IgM-rheumatoid factor (RF) with or without arthralgia symptoms predict the development of RA [1–4]. Early treatment of RA improves the outcome [5], and this principle may also apply to the preclinical phase of RA. Detecting patients with arthralgia at high risk of RA offers the opportunity to develop treatment strategies for prevention of RA in these patients. Current prediction rules for arthritis development based on clinical parameters (including autoantibodies) are suitable for this [4, 6–8], but their predictive value seems too low to ensure that all patients would be treated validly with medication with potentially serious side effects. The predictive capacity might be substantially improved by adding imaging [9].

Ultrasonography (US) is widely available at relatively low cost and has no radiation exposure. There is evidence that US increases diagnostic certainty when compared to clinical examination alone for diagnosing RA in early undifferentiated arthritis [10–15]. US was also described to add value to clinical examination in individuals at risk of developing RA [9, 11, 16–20], which may be particularly the case for power Doppler (PD) abnormalities [9, 11, 19] and mainly in autoantibody-negative persons [17, 18]. However, discrepancies related to the definition of US synovial thickness [21], the selection of joints included in the US protocol [21] and the use of different scoring systems [22–25] hamper general clinical implementation of US to help diagnose and predict RA [26].

In a previous study on the value of US in the prediction of arthritis in seropositive patients with arthralgia, we only scanned painful and adjacent/contralateral joints (which differed between patients) and showed that arthritis could be predicted at the joint but not at the patient level [16]. The present follow-up study included a new cohort of seropositive patients with arthralgia, in which we investigated the value of an US protocol including a standardized set of joints (regardless of local clinical symptoms) to predict clinical arthritis development. We also evaluated whether US abnormalities add predictive value to clinical parameters.

## Methods

### Study population

Seropositive patients with arthralgia (ACPA and/or RF), but without clinical arthritis, were recruited at Reade (Amsterdam) between March 2009 and December 2015. At the start of the study patients had very recently (0–2 weeks preceding inclusion) been evaluated by the treating rheumatologist who concluded they had arthralgia with autoantibodies and referred them to participate in the study. All had a new evaluation at study baseline where the study physician, who was trained in performing joint counts, collected data. In case of doubt about the presence of arthritis, another rheumatologist (involved in the study group) also performed the joint exam at this visit and made the final decision on presence or absence of arthritis. Patients with past arthritis or arthritis at baseline (defined as one or more swollen joints as reported by two independent investigators), age < 18 years and > 70 years, previous treatment with a disease-modifying anti-rheumatic drug or recent glucocorticoid treatment, systemic autoimmune disease, systemic infections, lymphoproliferative disorders or recent radiotherapy were excluded from the cohort [2, 16]. Medical history, tender joint count in 53 joints (TJC53), details of joint symptoms and ACPA/RF status were recorded at baseline [2], together with clinical criteria included in a previously described prediction rule for the development of arthritis in seropositive patients with arthralgia: presence of a first-degree relative with RA, alcohol consumption, symptom onset < 12 months, presence of intermittent symptoms, presence of symptoms in upper and lower extremities, presence of joint swelling (anamnestic), visual analog scale assessing pain (≥ 50 mm) and morning stiffness lasting at least 1 h [4]. These parameters (combined with the autoantibody status) were used to calculate a risk rule score ranging from 1 to 13, divided into three risk groups (low 0–4, intermediate 5–6, high 7–13). During yearly follow up, for up to 5 years, clinical arthritis development in any of 44 joints was assessed by a trained physician and an extra visit could be scheduled when arthritis development was suspected. If clinical arthritis was present in at least one joint, this was confirmed by a senior rheumatologist (DvS) without knowledge of the patient’s serostatus. The study was approved by the Slotervaart ziekenhuis and Reade ethics committee. Signed informed consent was obtained from all patients prior to inclusion.

### Ultrasonography

The joints were scanned according to a predefined standard US protocol of those 16 joints in which clinical swelling had developed most often in our previous US pre-RA cohort: bilateral wrists, metacarpophalangeal (MCP) 2–3, proximal interphalangeal (PIP) 2–3 and metatarsophalangeal (MTP) 2–3 and 5 [16]. All scans were performed using the Acuson Antares ultrasound system, premium edition (Siemens, Malvern, PA, USA) using linear array transducers VF 13–5 SP for

finger and toe joints (operating at 11.43 MHz for grayscale and 8.9 MHz for PD) and VF 13–5 for larger joints (operating at 11.43 MHz for grayscale and 7.3 MHz for PD), according to the manufacturer’s criteria [16]. The joints were scanned in the dorsal longitudinal plane from the most lateral to the most medial site and in the transverse plane from the proximal to distal site of the joint. Finger joints were also scanned in the palmar longitudinal plane. The wrist included scans of the radiocarpal and intercarpal joints and ulnocarpal joint including the ulnar styloid process. Effusion and synovial hypertrophy were scored in a combined measure (synovial thickening) as both phenomena often appear concurrently [27]. Synovial thickening and PD signs were scored using the four-grade semi-quantitative scale (0–3) of Szkudlarek [16, 22]. Synovial thickening grade ≥ 2 and PD grade ≥ 1 were regarded as abnormal. When multiple images were made of one joint, the highest score was used to obtain a single score per joint. US examinations were all performed by a single radiologist (MMR) experienced in musculoskeletal US, who was blinded to the clinical data. Ultrasound results were not available to the treating physician nor the physician performing follow-up study visits and thus the abnormalities did not change the way study participants were evaluated. Data were analyzed after all data were collected.

### Statistics

Normally distributed continuous data were summarized by the mean and standard deviation (SD). Non-normally distributed data were summarized by the median and interquartile range (IQR). The risk of arthritis development at the patient level was estimated by the chi-square or Fisher’s exact test, and corresponding positive and negative predictive values (PPV, NPV) were calculated. Results were expressed as odds ratios (OR) with 95% confidence interval (CI). Timing of arthritis development was assessed by Kaplan-Meier survival analysis using the log-rank test and Cox regression analysis, expressed as mean time to arthritis (we reported mean survival time instead of the mostly preferred median survival time, because in order to calculate the median, 50% of subjects need to develop arthritis and this was not the case in any of our groups) and hazard ratios (HR) with 95% CI. We also performed multivariate regression analysis to look at the additional value of US over clinical parameters in the patients with low, intermediate or high risk of developing RA [4]. Subgroup analyses were performed in ACPA-positive versus ACPA-negative patients. All analyses at the patient level were performed with and without inclusion of the MTP joints, as a previous study indicated that the frequency of synovial thickening in the MTP joints in healthy controls was too high to discriminate between those who will develop arthritis and those who will not [19]. The risk of arthritis development at the joint level (using all joints) was analyzed using generalized estimating equations (GEE) with an exchangeable correlation matrix, allowing correction for within-patient correlation [28]. Statistical analysis was performed using SPSS version 22 statistics software (SPSS Inc., Chicago, IL, USA).

## Results

In total, 287 seropositive patients with arthralgia were consecutively screened through our prospective cohort in the inclusion period. There were 14 patients excluded due to clinical arthritis at baseline, 99 patients did not receive US examination due to logistical problems or not consenting to US and 11 patients were lost to follow up after their baseline measurement. The remaining 163 patients were analyzed in the current study (74% female, mean ± SD) age 51 ± 11 years). Their baseline characteristics are shown in Table 1. Baseline characteristics of those who were included were similar to those who were not (data not shown). There were 51 patients (31%) who developed clinical arthritis after a median follow up of 12 (IQR 5–24) months: 44 patients (86%) who developed arthritis satisfied the 2010 American College of Rheumatology (ACR)/European League Against Rheumatism (EULAR) classification criteria for RA. The 112 patients who did not develop arthritis had a median follow-up time of 28 (IQR 19–49) months. US was performed within a median of 3 weeks (IQR 2–6 weeks) after the first visit. If we look at the distribution of pain among the 53 joints at baseline, we see that the shoulder was most often painful, followed by MTP3, acromioclavicular joint, sternoclavicular joint, MTP4, MTP2, wrist and knee. At the time of arthritis development, 6 out of 8 bilateral joints that were included in the ultrasound protocol (wrist, MCP2, PIP2, PIP3, MTP2 and MTP3) were among the 8 joints that were most often painful (together with shoulder and MTP4). Data on severity of pain were not available. There was no statistically significant association between pain and synovial thickening at baseline using GEE analysis (OR 2.1, CI 0.9–4.7, *p* = 0.08). When we excluded the MTPs from analysis we found association between pain and synovial thickening corrected for within-patient correlation (OR 3.5, CI 1.1–11.4, *p* < 0.05).

**Table 1:** Baseline characteristics

Baseline characteristics	Value in study population (*n* = 163)
Age in years, mean ± SD	51 ± 11
Female sex, *n* (%)	121 (74%)
Arthralgia duration in months, median (IQR)	13 (6–36)
Number of reported painful joints, median (IQR)	8 (4–19)
Tender joint count (53 joints), median (IQR)	1 (0–5)
VAS pain in mm (0–100), mean ± SD	35 ± 25
Antibody status	
ACPA negative, RF positive, *n* (%)	72 (44%)
ACPA positive, RF negative, *n* (%)	44 (27%)
ACPA positive, RF positive, *n* (%)	47 (29%)

*ACPA* anti-citrullinated protein antibodies, *IQR* interquartile range, *RF* rheumatoid factor, *SD* standard deviation, *VAS* visual analog scale

### US and clinical arthritis development at the patient level

At baseline, 49 patients (30%) had US synovial thickening and 7 patients (4%) had PD abnormalities in at least one joint (Table 2): of these, 5 patients (3%) had both synovial thickening and PD abnormalities in at least one joint and 3 patients (2%) had both synovial thickening and PD abnormalities in the same joint (with 1 patient having 4 joints with both synovial thickening and PD abnormalities). When excluding the MTP joints, 14 patients (9%) had synovial thickening in at least one joint and 7 patients (4%) had PD abnormalities.

**Table 2:** Association between ultrasound abnormalities and development of clinical arthritis/rheumatoid arthritis analyzed at the patient level

Ultrasound abnormalities	Arthritis, yes	Arthritis, no	OR (95% CI)	*p* value	PPV	NPV
Outcome: clinical arthritis	*n* = 51	*n* = 112				
Synovial thickening^a^ (16 joints)	19 (37%)	30 (27%)	1.6 (0.8–3.3)	0.18^c^	39%	72%
Synovial thickening (10 joints, no MTP)	10 (20%)	4 (4%)	6.6 (1.9–22.2)	< 0.01^d^	71%	72%
Power Doppler^a^ (16 joints)^b^	2 (4%)	5 (5%)	0.9 (0.1–4.7)	1.0.^d^	29%	69%
Outcome: 2010 RA criteria	*n* = 44	*n* = 119				
Synovial thickening (16 joints)	17 (39%)	32 (27%)	1.7 (0.8–3.6)	0.15^c^	35%	76%
Synovial thickening (10 joints, no MTP)	10 (23%)	4 (3%)	8.5 (2.4–28.7)	< 0.01^d^	71%	77%
Power Doppler (16 joints)^b^	2 (5%)	5 (4%)	1.1 (0.2–5.9)	1.0^d^	29%	73%

*CI* confidence interval, *MTP* metatarsophalangeal, *NPV* negative predictive value, *OR* odds ratio, *PPV* positive predictive value, *RA* rheumatoid arthritis

^a^Results are presented for synovial thickening and power Doppler in at least one joint

^b^Same results when excluding MTP joints

^c^Chi-square test

^d^Fisher’s exact test

Of the patients with US abnormalities in at least one joint, the median number of affected joints with synovial thickening was 2 (min-max 1–6) in the patients developing arthritis and also 2 (min-max 1–4) in the patients who did not develop arthritis. For PD abnormalities these numbers were 1 (1–5) and 3 (1–3), respectively.

A greater proportion of patients with US synovial thickening at baseline in at least one joint developed arthritis although this was not statistically significant (Table 2). This trend appeared to be more pronounced and was significant when the MTP joints were excluded (OR 6.6, CI 1.9–22.2, *p* < 0.01). The corresponding positive predictive value (PPV) and negative predictive value (NPV) were 71% and 72%, respectively. There was no statistically significant association between the presence of PD abnormalities in one or more joints and clinical arthritis development (OR 0.9, CI 0.1–4.7, *p* = 1.0, PPV 29%, NPV 69%). All patients with PD abnormalities in the MTP joints also had PD abnormalities in at least one other joint, therefore the association did not change when the MTP joints were excluded. Sensitivity analysis, with the development of RA defined according to the ACR/EULAR 2010 classification criteria as the outcome, showed a slight increase in the odds ratios and predictive values as compared to clinical arthritis as the outcome (OR 8.5, CI 2.4–28.7, *p* = < 0.01, PPV 71%, NPV 77%; Table 2).

**Fig. 1:**
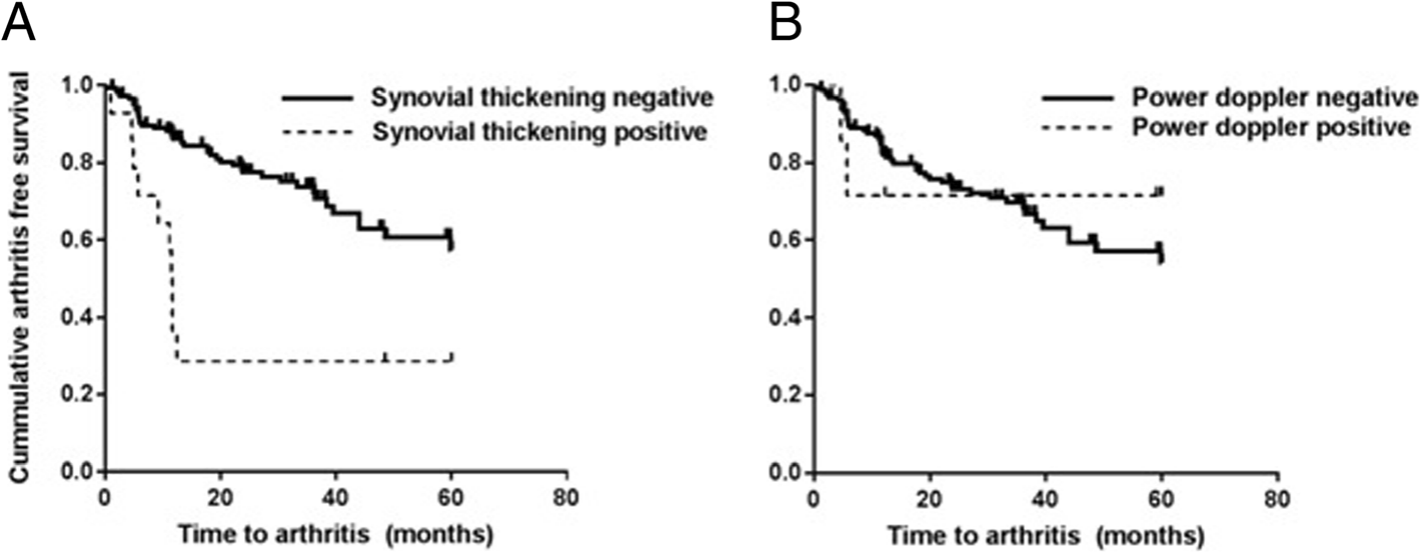
Kaplan-Meier curves for synovial thickening (**a**) and power Doppler (**b**) and time to arthritis development (months). The metatarsophalangeal joints were excluded

Clinical arthritis developed earlier in patients who had US synovial thickening in at least one joint than in those without US synovial thickening, but this was only the case when the MTP joints were excluded from the US assessment (mean time to arthritis 23 versus 45 months, *p* < 0.01; Fig. 1). The corresponding HR was 3.4 (CI 1.6–6.7, *p* < 0.01). Patients with PD abnormalities (both with and without the MTP joints included) did not develop arthritis earlier than patients without PD abnormalities (mean time to arthritis 44 versus 43 months, *p* = 0.7; Fig. 1) and the corresponding HR of 0.8 (CI 0.1–3.2, *p* = 0.7) was not statistically significant. In Fig. 1b the lines cross due to small numbers of PD-positive patients. Since this could be caused by effect modification, we investigated whether there was a significant difference between the effects in patients before and after a cutoff value of 30 months, but this was not the case.

We did not demonstrate clinically relevant differences in US abnormalities for prediction of arthritis at the patient level between ACPA-positive and ACPA-negative patients, except for synovial thickening without the MTP joints included in the ACPA-positive patients (19% developed arthritis versus 2% who did not; note the small numbers and thus wide CIs; Additional file 1: Table S1). The same was true for ACPA-positive versus ACPA-positive and RF-positive patients (Additional file 2: Table S2).

### US and clinical arthritis development at the joint level

US was performed at baseline on 2608 joints (16 joints per patient, no missing data). US revealed synovial thickening in 105/2608 (4%) of the joints, mostly in the MTP joints (84/105 (80%)). PD abnormality was seen in 14/2608 (0.5%) of the joints (of which 3/14 (21%) were in the MTP joints). In 158/2608 (6%) of the scanned joints at baseline, clinical arthritis developed during follow up (32% of this in MTP joints). At that time, the median number of joints with arthritis per patient was 3 (range 0–13, note that arthritis developed outside the standard set of 16 joints in 5 of the patients). Of the 158 joints with arthritis, US evidence of synovial thickening was only seen in the same joint at baseline in 8 joints (5.1%) (for PD this was 1 joint (0.6%)). There was no statistically significant association between the presence of synovial thickening in a joint and development of arthritis development in the same joint (OR 1.0, CI 0.3–2.9, *p* = 1.0) or between the presence of PD abnormality and development of arthritis in the same joint (OR 1.0, CI 0.2–4.8, *p* = 1.0) using GEE analysis.

### Added value of US for prediction of clinical arthritis versus clinical parameters alone

Patients were divided into three groups of low, intermediate and high risk of developing arthritis according to the clinical prediction rule score described in “Methods”. Multivariate regression analysis including the clinical prediction rule risk groups and synovial thickening in all joints excluding the MTP joints produced an OR of 6.1 (CI 1.6–23.2, *p* < 0.01) for synovial thickening and 3.5 (CI 2.2–5.5, *p* < 0.01) for the prediction rule groups. The number of patients in each group was too small to perform proper subgroup analysis, however, the relatively high OR of 6.1 for risk of synovial thickening seems to be due to both the patients from the intermediate risk group (in which four patients with synovial thickening developed arthritis and only one did not) and the high risk group (in which all six patients with synovial thickness also developed arthritis, see Table 3). For PD the OR in multivariate regression was 1.7 (0.3–10.2, *p* = 0.55), with an OR of 3.6 (2.3–5.6, *p* < 0.01) for the clinical score.

**Table 3:** Added value of ultrasound over clinical parameters according to a clinical prediction rule

Ultrasound abnormalities	Arthritis, yes	Arthritis, no	OR (95% CI)^d^	*p* value
Clinical prediction rule risk-groups^a^	*n* = 51	*n* = 112		
Synovial thickening^b^ (16 joints)			1.5 (0.7–3.4)	0.3
Clinical prediction rule			3.5 (2.2–5.4)	< 0.01
Low risk	2/8 (25%)	19/67 (28%)		
Intermediate risk	5/13 (38%)	5/25 (20%)		
High risk	12/30 (40%)	6/20 (30%)		
Synovial thickening (10 joints, no MTP)			6.1 (CI 1.6–23.2)	< 0.01
Clinical prediction rule			3.5 (CI 2.2–5.5)	< 0.01
Low risk	0/8 (0%)	3/67 (4%)		
Intermediate risk	4/13 (31%)	1/25 (4%)		
High risk	6/30 (20%)	0/20 (0%)		
Power Doppler^b^ (16 joints)^c^			1.7 (0.3–10.2)	0.5
Clinical prediction rule			3.6 (2.3–5.6)	< 0.01
Low risk	0/8 (0%)	4/67 (6%)		
Intermediate risk	2/13 (15%)	1/25 (4%)		
High risk	0/30 (0%)	0/20 (0%)		

*ACPA* anti-citrullinated protein antibody, *CI* confidence interval, *MTP* metatarsophalangeal, *OR* odds ratio, *RF* rheumatoid factor

^a^Risk groups based on the clinical prediction rule described in reference number 4

^b^Results are presented for synovial thickening and power Doppler in at least one joint (present, %)

^c^Same results when excluding MTP joints

^d^Logistic regression analysis (note that the prediction rule risk groups were combined)

## Discussion

Here we investigated whether abnormalities found with a standardized US protocol are useful in the prediction of arthritis development in seropositive patients with arthralgia, and whether these US abnormalities add predictive value over clinically available parameters. Synovial thickening on US (wrist and hand joints, excluding the MTPs) was associated with both arthritis development and its timing, at the patient level but not at the joint level. Also, US synovial thickening in the wrist and hand joints adds predictive value in patients with an intermediate-to-high risk of developing arthritis based on a clinical prediction rule. PD abnormalities on US were not associated with arthritis development.

The results should be interpreted in the light of small numbers of patients with US abnormalities, especially for PD. There were 31% of patients with abnormalities on US in at least one joint, which decreased to 10% when not analyzing the MTP joints. In total, only 4% presented with PD. Therefore, even in our population with a relatively high risk of developing arthritis (around 30%), a large number needs to be screened to find only a small proportion of patients with US abnormalities that progress to arthritis. This is undesirable for clinical implementation of US.

The usefulness of US as a predictor of arthritis or RA development has been described by several authors with varying results [9, 11, 16, 18, 19]. Our previous report of evaluating only painful joints concluded that presence of US abnormalities (both synovial thickness and PD) was associated with arthritis development at the joint level, but not at the patient level, which is opposite to our current conclusion [16]. A group from Leeds performed a study that included 136 ACPA-positive patients with musculoskeletal symptoms and showed that synovial thickening in two or more joints was related to 2.3 times greater chance of developing arthritis at the patient level, which increased to 3.7 for PD in at least 2 joints [19]. The HRs were even higher when analyzing on joint level (HR 9.4 for synovial thickening score ≥ 2 in a joint and HR 31 for PD ≥ 2). In another study, the same group found that PD signal added predictive value to clinical parameters in the prediction of arthritis [9] The higher scores (namely for PD) compared to our study may have been caused by the selection of patients with a higher a priori risk, as they were all ACPA-positive patients with arthralgia. A study from Switzerland focused on very early arthritis and evaluated 49 patients with inflammatory hand symptoms of recent onset (≤ 12 weeks) with or without clinical arthritis. Since all ACPA-positive and/or RF-positive patients eventually developed arthritis, the value of US was only determined in the seronegative patients [18]. In this subgroup the post-test probability in patients with 1–3 clinical parameters could be raised from 2–30% to 50–94% when using US synovial thickness or PD abnormality. Finally, synovial thickness (PD was not analyzed) was also researched in another seronegative patient population of 80, in which the OR of arthritis and/or RA development was 7.5 (clinical parameters were not taken into account) [18].

Three main reasons may have caused the different results presented above. First, different US scanning protocols were used. Our study in combination with our previous study indicates that applying a US protocol with a standardized set of joints results in better prediction at the patient level and that scanning only painful joints results in better prediction at the joint level. Although it appears attractive to scan more joints, both symptomatic and asymptomatic, this will make US more time-consuming and thus unfit for a clinical setting [29]. Also, it is still hard to define which set of asymptomatic joints should be used. In order to improve the selection of joints in the US protocol, future studies to determine in which joints ultrasound abnormalities are most predictive of arthritis development are needed. However, our study and a previous one has shown that it may be useful to exclude the MTP joints [19]. This would be convenient since it reduces the time taken to perform the US examination. The second reason for different results is technical differences between US machines, which mainly seems to be important for detection of the PD signal [30]. It is possible that in future studies, discrimination between effusion and synovial hypertrophy of the MTP joints may enhance prediction of those developing arthritis [31]; however, based on the available literature before the start of the study, effusion and synovial hypertrophy were believed to be part of the same pathophysiological process and were often seen combined, which was the reason for scoring them as one in the current study [27]. Differences between groups may be overcome in the future as the availability of good-quality US machines increases. The final reason is the fact that the use of US in prediction depends highly on the a priori chance of developing arthritis in the population that is investigated. In patients with an already high risk, for instance those who are ACPA-positive (for example, 42% developed arthritis in the Leeds cohort [19], 46% in the present study) or those with a high probability based on clinical prediction rules, having US abnormalities was almost always associated with arthritis development (6 patients in the present study, with a 100% chance of developing arthritis) [9, 17, 19]. However, US might be of even more value in those subpopulations of at-risk patients in which there is more diagnostic uncertainty, such as in seronegative patients with arthralgia [17, 18] and patients scoring intermediate on the clinical prediction rule. We did not include seronegative patients, but we did show that among the 5 patients with an intermediate risk of developing arthritis, 80% had US abnormalities.

Some additional comments can be made. First, it may be interesting to look not only at US abnormalities, but also to the absence of these in relation to a lower chance of developing arthritis. Van der Ven et al. [32], reported NPV of 89% for grayscale and/or PD abnormalities in 196 patients with inflammatory arthralgia. In the present study, the NPVs were somewhat lower for synovial thickness (72%) and PD abnormalities (77%) individually, although they were measured in a cohort with a low prevalence of US abnormalities. Second, it may be worthwhile to include tenosynovitis as an independent variable besides synovitis when looking at US abnormalities in the at-risk phase of RA [33]. Third, it was speculated that US is of greater value when applying the 2010 criteria for RA, because these criteria are designed to identify RA at an early stage [11]. This was confirmed by our study as both OR and NPV increased when the 2010 criteria for RA were used as the outcome measure. Last, a limitation of this study may be that US examinations were all performed by a single radiologist, although this radiologist had a high interobserver agreement between this radiologist and another was high in our previous study. [16].

## Conclusions

In conclusion, synovial thickening on US using a standard US protocol with exclusion of the MTPs predicted arthritis development and its timing in seropositive patients with arthralgia. PD did not predict arthritis development, probably related to low PD frequency. A large study population needs to be screened to find only a small percentage of patients with US abnormalities, so expected use for routine clinical practice and to select individuals at risk of developing arthritis for preventive studies is low. However, based on our data we do expect that US can be of additional use for clinicians in those patients who have an intermediate risk of developing arthritis when calculating the prediction rule, as compared to those patients for whom the risk is more clearly defined based on clinical parameters (low and high risk).

## Additional files

Additional file 1: **Table S1.** Association of ultrasound abnormalities with clinical arthritis development, ACPA-positive versus ACPA-negative patients (patient level). (DOCX 14 kb)

Additional file 2: **Table S2.** Association of ultrasound abnormalities with clinical arthritis development, only ACPA-positive versus ACPA-positive and RF-positive patients (patient level). (DOCX 15 kb)

## Abbreviations

ACPA: Anti-citrullinated protein antibodies
CI: Confidence interval
DvS: Dirkjan van Schaardenburg
GEE: Generalized estimating
HR: Hazard ratio
IQR: Interquartile range
MCP: Metacarpophalangeal
MMR: Marlies Meursinge Reynders
MTP: Metatarsophalangeal
NPV: Negative predictive value
OR: Odds ratios
PD: Power Doppler
PIP: Proximal interphalangeal
PPV: Positive predictive value
RA: Rheumatoid arthritis
RF: Rheumatoid factor
SD: Standard deviation
TJC53: Tender joint count 53
US: Ultrasonography/ultrasound
