# Supplementary material for: The value of joint ultrasonography in predicting arthritis in seropositive patients with arthralgia: a prospective cohort study
Source: Arthritis Res Ther. 2018 Dec 19;20:279. doi: 10.1186/s13075-018-1767-9 (PMC6300036; doi:10.1186/s13075-018-1767-9)
Supplement: Supplementary file 1 — Table S1. Association of ultrasound abnormalities with clinical arthritis development, ACPA-positive versus ACPA-negative patients (patient level). (DOCX 14 kb) [file 13075_2018_1767_MOESM1_ESM.docx]

**Table S1. Association of ultrasound abnormalities with clinical arthritis development, ACPA positive versus negative patients (patient level)**

| US abnormalities | Arthritis yes | Arthritis no | OR (95% CI) | p-value |
| --- | --- | --- | --- | --- |
| *ACPA positive patients* | n=42 | n=49 |  |  |
| Synovial thickening* (16 joints) | 16 (38%) | 14 (29%) | 1.5 (0.6-3.7) | p=0.4^†^ |
| Synovial thickening (10 joints, no MTP) | 8 (19%) | 1 (2%) | 11.3 (1.3-96) | p=0.01^‡^ |
| Power Doppler* (16 joints)** | 1 (2%) | 3 (6%) | NA | NA |
| *ACPA negative patients* | n=9 | n=63 |  |  |
| Synovial thickening (16 joints) | 3 (33%) | 16 (25%) | 1.5 (0.3-6.6) | p=0.6^‡^ |
| Synovial thickening (10 joints, no MTP) | 2 (22%) | 3 (5%) | 5.7 (0.8-40) | p=0.1^‡^ |
| Power Doppler (16 joints)** | 1 (11%) | 2 (3%) | NA | NA |
| ^†^ Chi-square test, ^‡^ Fisher’s exact test.  * Results are presented for synovial thickening and Power Doppler in at least one joint  ** Same results when excluding MTP joints  CI, confidence interval; MTP, metatarsophalangeal; OR, odds ratio; NA, not applicable (not calculated due to small numbers) | | | | |
