# Supplementary material for: The value of joint ultrasonography in predicting arthritis in seropositive patients with arthralgia: a prospective cohort study
Source: Arthritis Res Ther. 2018 Dec 19;20:279. doi: 10.1186/s13075-018-1767-9 (PMC6300036; doi:10.1186/s13075-018-1767-9)
Supplement: Supplementary file 2 — Table S2. Association of ultrasound abnormalities with clinical arthritis development, only ACPA-positive versus ACPA-positive and RF-positive patients (patient level). (DOCX 15 kb) [file 13075_2018_1767_MOESM2_ESM.docx]

**Table S2 Association of ultrasound abnormalities with clinical arthritis development, only ACPA positive versus ACPA-positive and RF-positive patients (patient level)**

| Ultrasound abnormalities | Arthritis yes | Arthritis no | OR (95% CI) | p-value |
| --- | --- | --- | --- | --- |
| *Only ACPA positive patients* | n=13 | n=31 |  |  |
| Synovial thickening* (16 joints) | 6 (46%) | 7 (23%) | 1.2 (0.2-7.5) | p=0.8^†^ |
| Synovial thickening (10 joints, no MTP) | 4 (31%) | 1 (3%) | 13 (0.8-210) | p=0.07^‡^ |
| Power Doppler* (16 joints)** | 1 (2%) | 2 (6%) | 0.5 (0.02-12.2) | p=0.7^‡^ |
| *ACPA and RF positive patients* | n=29 | n=18 |  |  |
| Synovial thickening (16 joints) | 10 (34%) | 7 (39%) | 0.6 (0.2-2.2) | p=0.4^†^ |
| Synovial thickening (10 joints, no MTP) | 4 (14%) | 0 (0%) | NA | NA |
| Power Doppler (16 joints)** | 0 (0%) | 1 (1%) | NA | NA |
| ^†^ Chi-square test, ^‡^ Fisher’s exact test.  * Results are presented for synovial thickening and Power Doppler in at least one joint  ** Same results when excluding MTP joints  ACPA, anti-citrullinated protein antibodies; CI, confidence interval; MTP, metatarsophalangeal; NA, not applicable (not calculated due to small numbers); OR, odds ratio; RF, rheumatoid factor | | | | |
